# Unique Associations between Insulin-Like Growth Factor Binding Protein-1, Insulin-Like Growth Factor-1 and T Cell Immunoglobulin Mucin 3 in Successful Twin Pregnancies Conceived with Donor Oocytes

**DOI:** 10.3390/medicina55050144

**Published:** 2019-05-16

**Authors:** Giovanni Sisti, Mariarosaria Di Tommaso, Sara Paccosi, Astrid Parenti, Viola Seravalli, Roberta Cuzzola, Steven S. Witkin

**Affiliations:** 1 Department of Obstetrics and Gynecology, Lincoln Medical and Mental Health Center, The Bronx, NY 10451, USA; 2 Department of Obstetrics and Gynecology, Weill Cornell Medicine, New York, NY 10065, USA; switkin@med.cornell.edu; 3 Department of Health Sciences, Obstetrics and Gynecology Branch, University of Florence, 50134 Florence, Italy; mariarosaria.ditommaso@unifi.it (M.D.T.); viola.seravalli@unifi.it (V.S.); roberta.cuzzola@gmail.com (R.C.); 4 Department of Health Sciences, University of Florence, 50134 Florence, Italy; sara.paccosi@unifi.it (S.P.); astrid.parenti@unifi.it (A.P.); 5 Institute of Tropical Medicine, University of Sao Paulo Medical School, Sao Paulo 05403-000, Brazil

**Keywords:** donor oocytes, insulin-like growth factor-1, insulin-like growth factor binding protein-1, in vitro fertilization, pregnancy outcome, T cell immunoglobulin mucin-3

## Abstract

*Background and Objectives*: To investigate if pregnancies conceived using an oocyte donor necessitate an alteration in immune regulation, we compared concentrations of insulin-like growth factor binding protein (IGFBP)-1, insulin-like growth factor (IGF)-1 and T cell immunoglobulin mucin-3 (Tim-3) in women with ongoing successful twin pregnancies conceived spontaneously, using assisted reproductive technologies that utilized homologous oocytes or with donor oocytes. Differences in levels of these immune modulatory proteins may be magnified and easier to detect in twin as compared to singleton pregnancies. *Methods*: In this prospective study IGFBP-1 and IGF-1 were measured in sera and Tim-3 in lysates of peripheral blood mononuclear cells (PBMCs) by ELISA. *Results*: Median IGFBP-1 levels were lower in women with donor oocytes (41.4 ng/ml) as compared to those with a spontaneous conception (51.2 ng/mL) or who conceived with various assisted reproduction protocols using homologous oocytes (52.4 ng/mL) (*p* < 0.001). IGF-1 and Tim-3 levels were comparable in each group. The IGFBP-1 level was inversely correlated to the IGF-1 concentration only in women with donor oocytes (*p* = 0.032). IGFBP-1 and Tim-3 levels were similarly negatively correlated in the donor oocyte group (*p* = 0. 012). Women in the assisted reproduction group who conceived following intracytoplasmic sperm injection were the only other group in which IGFBP-1 and Tim-3 were negatively correlated (*p* = 0.018). *Conclusions*: Down-regulation of IGFBP-1 production in pregnancies conceived with donor oocytes may reduce the extent of pro-inflammatory immunity and contribute to successful outcome in totally allogeneic pregnancies.

## 1. Introduction

Pregnancies involving an oocyte donor differ from conventional gestations. In the absence of a corpus luteum, the post-conception rise in estrogen and progesterone do not occur and these essential hormones have to be exogenously supplied [1]. Additional compounds normally released from an activated ovary also fail to be produced [2]. In conventional pregnancies, the maternal immune system must respond to a fetus that expresses antigens derived from foreign paternal chromosomes (semi-allogeneic). However, when fetuses are derived from fertilization of oocytes from a third-party oocyte donor and then implanted in a second woman, both paternal and maternal antigens expressed by the embryo are foreign. This places an additional burden on the maternal immune system to effectively regulate the extent of reactivity to antigens from two exogenous sources. As a result, immune-mediated placental pathology, has been shown to occur more frequently after pregnancy with oocyte donors [3,4] and there is evidence of an increase in both pro- and anti-inflammatory immune activity [5]. The most definitive study to date on maternal immune responses to donor oocytes identified several differences from conventional pregnancies [6]. Cytokines levels in peripheral blood and in the placenta are altered, and the number of activated T lymphocytes are elevated. As a probable consequence of this elevated burden placed on the maternal immune system by the presence of a totally allogeneic fetus the risk to develop preeclampsia is highest in donor oocyte gestations [7].

One mechanism that influences the magnitude of immune responses is an alteration in production and/or activity of insulin-like growth factor binding protein-1 (IGFBP-1) and insulin-like growth factor-1 (IGF-1). IGF-1 binds to cell surface receptors and induces the induction of pro-inflammatory immunity [8]. IGFBP-1 competes for IGF-1 binding to cell surface receptors and down-regulates IGF-1-directed inflammation [8]. In addition, IGFBP-1 produced in the decidua during the initiation of embryo implantation [9] interacts with IGF-1 and facilitates placental development by regulating the extent of trophoblast migration at the maternal-fetal interface [9]. IGFBP-1–IGF-1 interactions also regulate the extent of fetal growth during early pregnancy [10,11].

Another mechanism to regulate immune activity is by the action of immune checkpoint inhibitors. T cell immunoglobulin mucin 3 (Tim-3) is one such inhibitor located on the surface membrane of activated T lymphocytes, macrophages/monocytes and natural killer cells [12,13,14]. Upon activation Tim-3 suppresses pro-inflammatory immunity and thus, prevents prolonged immune activation [15]. During semi-allogeneic pregnancy, Tim-3 expression is up-regulated in peripheral blood cells [16] and results in down-regulation of pro-inflammatory immunity at the maternal-fetal interface [17]. Both an elevation or an inhibition in Tim-3 expression beyond optimal levels are associated with pregnancy failure and spontaneous abortion [16,17,18].

In this communication, we evaluated whether variations in expression of IGFBP-1, IGF-1 and Tim-3 and their interactions, differed between twin pregnancies that occurred spontaneously, resulted from assisted reproductive technologies that utilized homologous oocytes or that employed donor oocytes. We envisioned that changes in immune system regulation, perhaps difficult to observe in singleton pregnancies, would be amplified and more readily demonstrable in multifetal pregnancies due to the doubling of the number of fetuses.

## 2. Materials and Methods

### 2.1. Subjects

The study population in this prospective study consisted of 112 women with dichorionic diamniotic twin pregnancies attending the outpatient obstetrics clinic at Careggi University Hospital in Florence (Italy). Subjects were divided into three groups according to the mode of conception. Group 1 was composed of 43 women with spontaneous conceptions; group 2 was 44 women who conceived by assisted reproduction, either merely ovarian stimulation with clomiphene citrate (3), intrauterine insemination (3), in vitro fertilization and embryo transfer (IVF-ET) (20), intracytoplasmic sperm injection (ICSI) (18); group 3 was 25 women who were pregnant by IVF using oocyte donation. Samples were collected in mid-pregnancy, late second to early third trimester. Exclusion criteria included the presence of a fetal malformation, pregnancy complication such as chronic hypertension, diabetes, gestational hypertension, intra-uterine growth restriction of one or both fetuses, cholestasis of pregnancy or inability to provide informed consent. The study was approved by the Institutional Research Ethics Board at Careggi Hospital (30 May 2017, with number 10255_bio) and all subjects provided informed written consent.

Following completion of the laboratory study demographic characteristics, medical and obstetrical history, information regarding current pregnancy including gestational age at sample collection, number of fetuses, complications during pregnancy (including presence of gestational diabetes and use of betamethasone for fetal lung maturation) and gestational age at delivery were obtained by chart review. Patient who had a preterm iatrogenic delivery were excluded from the gestational age at delivery statistical analysis. Pregnancy dating was performed according to last menstrual period or crown-rump length (CRL) recorded at ultrasound in the first trimester.

### 2.2. Assays

Blood samples were obtained from a peripheral forearm vein at the time of a routinely scheduled check-up into a heparinized tube. Peripheral blood mononuclear cells (PBMCs) were isolated by Ficoll-Hypaque density gradient centrifugation, resuspended to a concentration of 5 million cells per ml and 0.1 mL aliquots were immediately lysed in a detergent solution containing protease inhibitors, as previously described [19]. The lysates were centrifuged and the supernatants were immediately frozen in aliquots at −80 °C. Unheparinized blood was also obtained at the same time. The serum fraction was collected by centrifugation and frozen in aliquots at −80 °C. All sera and PBMC lysates were shipped in a single batch on dry ice to the Witkin lab for analysis. All samples remained frozen during shipment. Thawed serum aliquots were assayed in duplicate for concentrations of IGFBP-1 and IGF-1 by commercial ELISA kits (R&D Systems, Minneapolis, MN, USA) by investigators blinded to all clinical data. Similarly, Tim-3 in the lysates was measured by ELISA (R&D Systems). Values were converted to ng/ml by reference to a standard curve that was generated in parallel to each assay. The lower limit of sensitivity was 37 pg/mL for IGFBP-1, 31 pg/mL for IGF-1 and 8.8 pg/mL for Tim-3.

### 2.3. Data Analysis

All the continuous variables were checked for normality with the Shapiro-Wilk test and resulted not-normally distributed. The non-parametric Kruskal-Wallis test was used to compare continuous variables while the chi-square test was used to compare categorical variables. The Spearman rank correlation test evaluated associations between IGF-1, IGFBP-1 and Tim-3. The statistical analysis was performed using SPSS version 21.0. Differences were considered significant when the *p* value was <0.05. 

## 3. Results

Characteristics of the study population are shown in Table 1. The women who conceived using donor oocytes were significantly older than women in the other two groups (*p* < 0.001) and had the lowest parity (*p* < 0.001). The median gestational age at sample collection, body mass index, use of betamethasone for lung maturation, presence of gestational diabetes, gestational age at delivery, delivery by cesarean section, the birthweight of both babies, smoking history and percent of White race were comparable in all three groups. 

**Figure 1:**
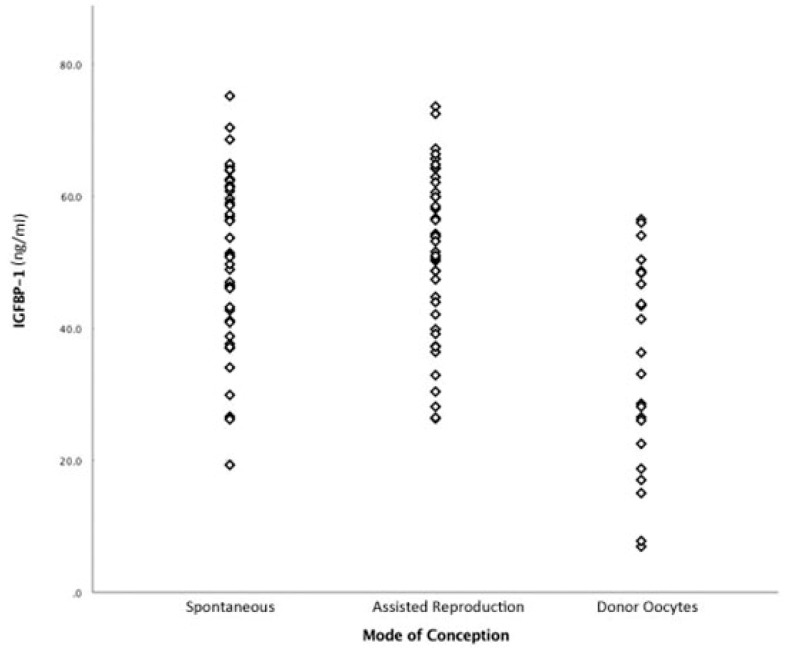
Serum insulin-like growth factor binding protein (IGFBP)-1 levels in women with twin pregnancies that were conceived spontaneously, employing assisted reproduction technologies using homologous oocytes or that utilized donor oocytes. Sera from women in the three groups were tested for levels of IGFBP-1 by ELISA.

The concentrations of IGFBP-1 and IGF-1 in the sera and Tim-3 in the PBMC lysates, from women in the three groups are shown in Table 2. The median levels of IGF-1 and Tim-3 were comparable regardless of the mode of conception and utilization of the mothers’ or donors’ oocytes. In contrast, the median level of IGFBP-1 was significantly lower in women who were pregnant with donor oocytes (*p* = 0.001). Individual values for all study subjects are shown in Figure 1. Similar values for all three compounds were obtained when subjects in group two were separated by the specific assisted reproduction technique that was performed (data not shown). There were no associations between the serum IGFBP-1 level and maternal age, parity or time since initial sample collection in Italy. 

Associations between concentrations of IGFBP-1, IGF-1 and Tim-3 in each of the three subject groups are shown in Table 3. Serum concentrations of IGF-1 and IGFBP-1 were negatively correlated (Spearman r = −0.431, *p* = 0.032) only in women with pregnancies that utilized donor oocytes. The level of IGFBP-1 in the circulation was also negatively associated with the Tim-3 concentration in PBMCs from women in the donor oocyte group (Spearman r = 0.012, *p* = 0.012), as well as in the subpopulation of women in the assisted reproduction group who became pregnant following ICSI (Spearman r = −0.552, *p* = 0.018). There were no associations between Tim-3 and IGF-1 in any of the three groups.

## 4. Discussion

We identified a reduction in the concentration of IGFBP-1 in the circulation of women with twin pregnancies derived from donor oocytes as compared to IGFBP-1 levels in women with twin pregnancies from spontaneous conceptions or from assisted reproduction technologies that utilized homologous oocytes. This finding is consistent with a previous report of reduced serum IGFBP-1 levels in singleton ovum donor-related pregnancies [1]. In the singleton study, it was suggested that an absence of ovarian stimulation in women with donor oocytes resulted in a loss of IGFBP-1 and/or IGFBP-1 inducers that would normally be released from ovarian tissues in gestation. We further show that the IGFBP-1 level is inversely correlated to the IGF-1 level only in those twin pregnancies that employed donor oocytes and that IGFBP-1 and Tim-3 concentrations are also inversely correlated in women who employed donor oocytes. 

IGFBP-1 interacts with IGF-1 and reduces its bioavailability [11]. The strong negative association between serum levels of these two compounds only in twin pregnancies that utilized an oocyte donor suggests that a similar relationship might also exist at the maternal-fetal interface. This would result in a more stringent control over trophoblast migration, placental development, pro-inflammatory immune activation and the rate of fetal growth than would occur in semi-allogeneic pregnancies. In pregnancies with homologous oocytes, there may be less of a need for a more precise coordination of IGFBP-1 and IGF-1 production, due, perhaps, to the presence of additional immune regulatory factors emanating from the ovary. The negative association between serum IGFBP-1 and Tim-3 expression on PBMCs also supports the occurrence of unique regulatory mechanisms that coordinate immune activity in twin gestations derived from donor oocytes. 

We also observed a negative association between IGFBP-1 and Tim-3 levels in women who became pregnant via ICSI but not in those who utilized IVF-ET. This suggests that the state of the ovary, in addition to regulating IGFBP-1 production, may also exert an influence on Tim-3 expression on lymphoid cells. The assisted penetration of the oocyte by the sperm may induce changes in properties of fertilized ova that influence immune checkpoint inhibitor expression during pregnancy. This unexpected observation deserves further study. 

Our study has several limitations. Serum pro- or anti-inflammatory cytokines were not measured and so there is no direct evidence relating the IGFBP-1 concentration to the cytokine level in the circulation of women with donor oocyte-related pregnancies. While it would have been advantageous to include these measurements, their analysis would not necessarily directly relate to the state of immunity at the maternal-fetal interface which differs from that present in the peripheral circulation [6]. In addition, other possible influences on immune regulation such as circulating levels of insulin [20] or IGF-2 [21] were not evaluated in our study. Although we did utilize a control group that underwent IVF using homologous oocytes, it cannot be definitively ruled out that conditions unique to the collection, incubation and transfer of donor oocytes had an unknown effect on IGFBP-1 production. Another limitation is the smaller number of women in the donor oocyte group compared to subjects in the other groups. However, we wish to stress that an immune evaluation of women with multifetal gestations who conceived using donor oocytes has not been reported previously to our knowledge and so dissemination of our findings, although not definitive, is hopefully of sufficient interest to spur additional investigations in this area. It should be noted that our study was confined to women with successful ongoing pregnancies and deliveries. Whether deviations from these relationships are associated with failed donor oocyte-related pregnancies remain to be determined.

Age and parity are significantly different in the oocyte recipient group compared to others. These two variables could have influenced the levels of IGFBP-1 and this is a limitation of the study; however, this is an invariable issue with IVF studies, since IVF patients are usually older and nullipara, compared to spontaneous conception patients, who usually are younger and multipara.

## 5. Conclusions

In conclusion, compared to those with twin gestations derived from their own oocytes, women with twin pregnancies conceived using donor oocytes have reduced circulating levels of IGFBP-1 and the concentration of IGFBP-1 is coordinated with levels of IGF-1 and Tim-3.

## Figures and Tables

**Table 1:** Characteristics of the study population.

Characteristic	Natural Conception	Assisted Reproduction	IVF-Donor Oocytes
	*n* = 43	*n* = 44	*n* = 25
Age in years	34 (31–36)	35 (33–38.7)	42 (37–45) ^b^
BMI (kg/m^2^)	21.1 (19.9–23.9)	21.4 (20.2–23.1)	23.3 (19.9–28.2)
Parity >0	51.2%	6.8%	4% ^b^
Betamethasone	32.5%	31.8%	16%
Gestational Diabetes	18.6%	11.3%	28%
Cesarean section	97%	90%	95%
Gestational age sample ^a^	24 (19–28)	19 (16–26)	21 (19–26)
Gestational age delivery ^a^	37 (34–37)	37 (35.5–38)	36 (35–37)
Birthweight 1st baby	2430 (1930–2550)	2530 (2190–29,000)	2390 (1950–2662)
Birthweight 2nd baby	2290 (2140–2650)	2420 (2090–2710)	2230 (1867–2655)
Smoking	9.3%	0	4%
White race	93%	95.5%	96%

Numbers in parenthesis are standard deviation; ^a^ median (range) weeks; ^b^ *p* < 0.001; IVF: in vitro fertilization; BMI: body mass index.

**Table 2:** Insulin-like growth factor binding protein-1 (IGFBP-1), insulin-like growth factor-1 (IGF-1) in sera and T cell immunoglobulin mucin-3 (Tim-3) in peripheral blood mononuclear cells (PBMCs) from women with twin pregnancies.

Group	Median (Range)		
	IGFBP-1 ^a^	IGF-1 ^a^	Tim-3 ^b^
Spontaneous conception	51.2 (41.1–61.4)	2.4 (1.1–3.9)	201 (132–288)
Assisted reproduction	52.4 (39.3–61.7)	2.5 (1.4–4.5)	215 (159–263)
Donor oocytes	41.4 (24.2–48.6) ^c^	2.7 (1.5–4.3)	197 (155–287)

^a^ ng/mL; ^b^ pg/mL; ^c^ *p* = 0.001; IGFBP-1: Insulin-like growth factor binding protein-1; IGF-1: Insulin-like growth factor-1; Tim-3: T cell immunoglobulin mucin-3.

**Table 3:** Associations between IGFBP-1, IGF-1 and Tim-3.

Pair	Group	Spearman r	*p* Value
IGFBP-IGF-1	Spontaneous	NS	NS
	Assisted reproduction	NS	NS
	Donor oocytes	−0.431	0.032
IGFBP-1-Tim-3	Spontaneous	NS	NS
	Assisted reproduction ^a^	−0.552	0.018
	Donor oocytes	−0.495	0.012
IGF-1-Tim-3	Spontaneous	NS	NS
	Assisted reproduction	NS	NS
	Donor oocytes	NS	NS

^a^ This association was confined to the subgroup of women who underwent ICSI followed by IVF. There was no association between IGFBP-1 and Tim-3 in the other subgroups of women in this group. NS: not significant
